# Yeast-Based Direct Catalytic Ethanol Fuel Cell Biosensors: A Batch Analysis Apparatus Combined with Chemometrics for Qualitative Carbohydrate Detection

**DOI:** 10.3390/bios15020096

**Published:** 2025-02-08

**Authors:** Mauro Tomassetti, Federico Marini, Corrado Di Natale, Mauro Castrucci, Luigi Campanella

**Affiliations:** 1 Department of Chemistry, University of Rome “La Sapienza”, P.le A. Moro 5, 00185 Rome, Italy; mauro.castrucci@libero.it (M.C.); luigi.campanella@uniroma1.it (L.C.); 2 Department of Electronic Engineering, University of Rome “Tor Vergata”, Via del Politecnico 1, 00133 Rome, Italy; dinatale@uniroma2.it

**Keywords:** carbohydrates, qualitative analysis, direct catalytic fuel cell (DCFC), chemometrics, common dimension (ComDim)

## Abstract

A novel strategy for the qualitative analysis of carbohydrates is developed, utilizing a direct catalytic fuel cell (DCFC) as a sensor, combined with chemometric tools for processing the resulting response curves. Specifically, carbohydrate solutions were incubated with yeast to produce alcohol, and the corresponding current decay trends were measured using a direct catalytic fuel cell designed for ethanol detection. Multiple data processing approaches were then evaluated. Initially, the entire set of data points from the response curves was analyzed using principal component analysis (PCA). To reduce analysis time, chemometric processing was subsequently restricted to the initial portion of the response curves. Finally, to enhance the results, the current decay curves were analyzed in conjunction with the linear fitting parameters derived from the quasi-linear region of the initial response curves, utilizing the common dimension (ComDim) algorithm.

## 1. Introduction

For many years, sensors—particularly electrochemical sensors—have been widely recognized, valued, and extensively used as part of established analytical methods [1,2,3]. These sensors and biosensors are routinely employed, primarily for quantitative purposes, to determine various organic substances, as well as inorganic cations and anions. However, their potential qualitative analytical applications are rarely explored, with a few notable exceptions, such as electrochemical immunosensors and, in certain cases, enzyme-based sensors [4] or molecularly imprinted polymers applied to electrochemical sensors and electronic tongues [5].

Even in such cases, the focus remains predominantly on their quantitative applications, despite the fact that the high selectivity (or specificity) of these devices could, in principle, enable their use for the qualitative identification of specific substances. Nonetheless, the emphasis continues to be placed on quantitative applications, which are more widely proposed, prioritized, and valued, while their qualitative potential is largely overlooked.

The limited use of conventional electrochemical sensors and biosensors for qualitative analysis is understandable. These sensors typically rely on the detection of specific functional groups, such as alcohol, phenolic, or amino groups, and thus, they are generally unable to distinguish between compounds containing the same functional group. For example, while a sensor may exhibit a stronger response to one phenol compared to another, it only provides a general “pool” measurement in the presence of multiple compounds sharing the same functional group [6]. On the other hand, the limited adoption of highly specific or selective sensors for qualitative analysis—such as electrochemical immunosensors—seems less justified. These devices are capable of offering high specificity, yet even in such cases, quantitative analysis is favored. The primary reason for this bias lies in the operational format of these sensors. Electrochemical sensors must be immersed in a solution containing the target species for measurement, a seemingly straightforward process that introduces several challenges to the repeatability and reliability of the signal.

To ensure identical and reproducible signals, even for the same chemical species, factors beyond the concentration of the analyte must be controlled. These include the depth of sensor immersion, as variations in hydrostatic pressure can influence the response. Additionally, solutions often require consistent stirring, and reproducing such conditions precisely can be difficult. The specific location within the solution where the sensor is immersed can also affect the response, as fluid dynamics may vary even within closely spaced regions, resulting in different stirring speeds or flow patterns.

While these factors may introduce variations, they are usually negligible in quantitative analysis, where concentration differences have a much greater influence on the sensor’s response. Repeated measurements and averaging further mitigate these effects. However, in qualitative analysis, these variations can become more significant, especially when the difference in sensor response between functional groups in different compounds is inherently small. In conclusion, for traditional electrochemical sensors, regardless of their type, these operational challenges—though often minor—can pose significant obstacles to qualitative analysis. The inherent limitations of the measurement format make it difficult to achieve reliable differentiation between compounds, even with highly selective sensors.

The authors of this paper recently conceived an idea that, if feasible, could potentially eliminate the drawbacks associated with the conventional measurement process using electrochemical sensors. The concept involves completely reversing the geometry of the measurement setup. Instead of moving the sensor into and out of the solution, the sensor would remain stationary, while the solution is transferred into the sensor itself. This approach would allow the electrochemical measurement to be conducted only after the solution has fully stabilized. Upon further investigation, the authors realized that such devices already exist—referred to as electrocatalytic fuel cells operating in batch mode—and that these could be utilized by processing their entire response signal using chemometric techniques.

In previous research, the authors employed fuel cells to quantify alcohol and glucose content in beverages, drinks, and pharmaceutical products [7,8,9], focusing exclusively on quantitative analytical applications. However, in more recent studies, they explored the potential of direct catalytic ethanol fuel cells (DCEFCs) for qualitative identification as well. For instance, prior work [10] demonstrated the differentiation of aliphatic alcohols and other molecules by employing suitable chemometric methods to analyze the entire current response of the DCEFC.

In the current study, the same strategy was extended to qualitatively differentiate several carbohydrates, including glucose, xylose, fructose, galactose, and sucrose. To facilitate batch measurements using the fuel cell, the carbohydrates were incubated with yeast in a suitable apparatus (previously used for quantitative analysis [11]) to produce the corresponding alcohols. These alcohols were then completely oxidized to water and CO_2_ in the anodic compartment of the catalytic fuel cell. The oxidation process occurred at different rates (i.e., varying response times) and sensitivities for different aliphatic alcohols, particularly those with longer carbon chains [10].

## 2. Materials and Methods

### 2.1. Incubation and Measurement of Response Curves

**Figure 1:**
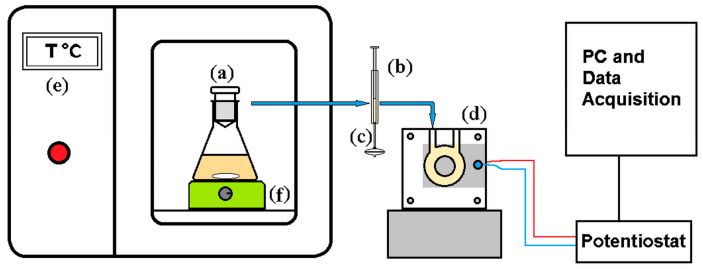
Schematic representation of the yeast incubation–fuel cell system operating in batch mode: (**a**) glass vessel for yeast–glucose incubation under stirring, (**b**) system for transferring the resulting alcohol solution into the fuel cell (syringe), (**c**) appropriate filter, (**d**) fuel cell, (**e**) thermostat, and (**f**) magnetic stirrer. The red and blue lines indicate respectively the cables that connect the positive and negative pole of the Fuel Fell to the potentiostat.

Incubation (12 h) was conducted using 0.6 g of yeast, in a glycine buffer, at a temperature of 28.5 °C. All carbohydrate samples were analyzed at a uniform concentration of 0.03 mol L^−1^. At the end of the incubation period, 2 mL of the solution was promptly extracted from the incubation flask using a device consisting of a graduated syringe equipped with two small filters. This extracted solution was then injected into the direct catalytic ethanol fuel cell (DCEFC) (Figure 1).

**Figure 2:**
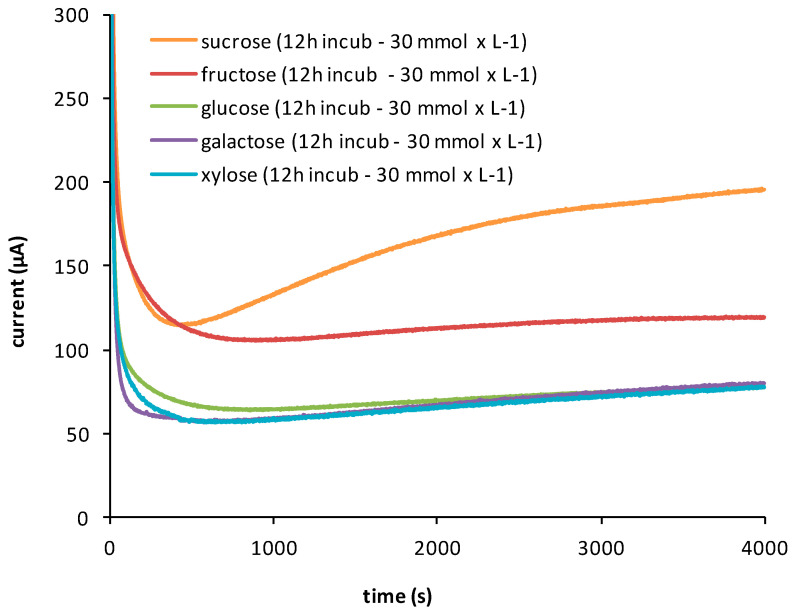
Each curve shown in this figure represents the complete current response profile for the five carbohydrates investigated, measured up to the steady state. For each trend, at least three replicate determinations were performed to verify the repeatability of the response for all carbohydrates. The concentration of each carbohydrate during incubation, as described in Section 2.2, was consistently maintained at 30 mmol L^−1^. Incubation was conducted at 28.5 °C for 12 h. The subsequent response curves represent the measurement of the alcohol produced during incubation, as recorded by the fuel cell.

The fuel cell operated in potentiostatic mode, with the response current—referred to as the supplied current—measured using an EmStat potentiostat from Palmsens (Houten, The Netherlands), which was connected to the DCEFC. The electrode of the DCEFC was composed of a Pt-Ru black catalyst integrated with a Nafion™ membrane. The initial analytical strategy involved recording the complete current response profiles for the five carbohydrate molecules under investigation (see Figure 2).

### 2.2. Materials

The five carbohydrates (glucose, xylose, fructose, galactose, and sucrose), along with glycine for the buffer solution, were all of analytical grade and purchased from Sigma-Aldrich (St. Louis, MO, USA).

All solutions used to record the response curves were prepared by dissolving the appropriate amounts directly in a precisely measured volume of distilled and deionized water (conductivity: 0.01–0.02 µS). Specifically, 0.34 g of glycine (Fluka, Seelze, Germany; assay > 99%) was dissolved in 30 mL of water to create an isotonic medium (0.15 mol L^−1^, pH = 6.0) containing 0.6 g of yeast (*Saccharomyces cerevisiae*).

Each carbohydrate was accurately weighed using an analytical balance (Mettler PM460, Columbus, OH, USA) to achieve a final concentration of 30 mmol L^−1^ in the glycine–yeast solution. The yeast used for incubation was a commercially available product, purchased from a local supermarket (Conad, Rome, Italy) [11].

### 2.3. Apparatus

A direct catalytic fuel cell for methanol or ethanol (DC (M or E) FC), H-TEC Model F111, with dimensions of 50 × 50 × 40 mm and a weight of 100 g, was obtained from Fuel Cell Store (College Station, TX, USA). The cell was made in Plexiglas^®^, with an electrode end plate made of a Pt-Ru black catalyst, assembled with a Nafion™ membrane. For potentiostatic measurements, a Palmsens EmStat potentiostat (Houten, The Netherlands) was used, connected to the fuel cell, and interfaced via PSTrace Software version 4.6 on a Compaq Presario PC. During each measurement, the current was continuously recorded until a steady state was reached for qualitative analysis.

To achieve this, the current values at all points along the discharge curves were recorded and initially used for qualitative assessment. The final steady-state current values for different concentrations of the same fuel were subsequently used for potential quantitative analysis. The fuel cell was connected to the EmStat, with the anode serving as the working electrode, while the cathode functioned as both the reference and counter electrode. Before recording current measurements, EmStat automatically measured the open circuit voltage (OCV) for approximately 200 s. The anode potential was then set to the optimized applied potential (OAP), defined as OCV −100 mV [7,8,9,10,11,12,13,14].

### 2.4. Measurements

Figure 1 illustrates the experimental setup used for batch measurements. A 50 mL glass flask, sealed with a glass stopper and filled with 30 mL of a yeast–glucose–glycine solution, was maintained at a constant temperature of 28.5 °C for 12 h. The yeast suspension was continuously stirred at 100 rpm using a magnetic stirrer. At the end of the incubation period, a 2 mL aliquot was rapidly extracted from the flask using a graduated syringe fitted with two small filters (see Figure 1) and injected into the DCFC for analysis.

### 2.5. Chemometric Methods

The qualitative identification of the analytes relies on the use of single- and multi-block exploratory techniques. Single-block data processing (i.e., the elaboration of individual data matrices) was carried out via principal component analysis (PCA) [15,16,17,18], which is by far the most well-known and widely used chemometric method. The technique relies on compressing the information in data matrix X through the calculation of a set of latent variables (linear combination of the original ones) called principal components (PCs). Mathematically, this is accomplished by bilinearly decomposing the data matrix **X** as follows:

**X = TP^T^ + E**

where T is the matrix collecting the coordinates of the samples along the principal components (scores) while the loadings, i.e., the cosines defining the orientation of the latent variables in the original variable space, are gathered in matrix **P**. The fraction of the variability in the original data not accounted for by the PCA model is then collected in residual matrix **E**.

When more than a single block of data, i.e., more than a single matrix, is collected to describe the same set of samples, data analysis can profit from the integration of the information from the different data blocks through data fusion approaches. In the context of exploratory data analysis, one of the most popular tools for performing data fusion is the method called common components and specific weight analysis (CCSWA), better known as the common dimension (ComDim) algorithm [19,20,21]. As the name suggests, ComDim focuses on extracting components that describe the information content, which is common among the different blocks, so it assumes that the same set of scores can be used to capture the relevant variability in the different data matrices. At the same time, a particular component will account for a different fraction of the variance in the different blocks: The fraction of the variance of a particular block explained by a specific common component is called its salience. Accordingly, if a component has comparable salience for all blocks, it is a truly common one; on the other hand, if it has high salience only for one block, it mostly describes the variability in that particular data matrix.

## 3. Chemometric Elaboration and Results

**Figure 3:**
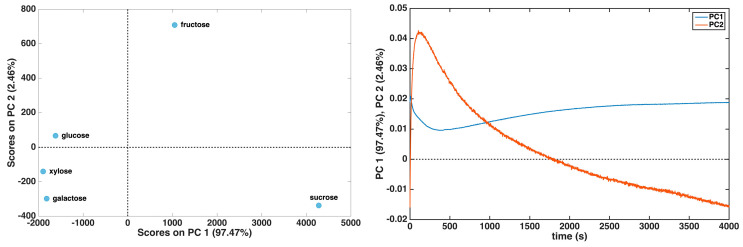
PCA results for the dataset comprising complete current response curves up to 4000 s: score plot (**left** panel); loading plot (**right** panel).

At first, principal component analysis was applied to the dataset made up of whole current response curves (reported in Supplementary as an Excel file) after mean centering. The resulting score and loading plots for the first two principal components, accounting for more than 99% of the original variance, are both reported in Figure 3 (left and right panels, respectively).

The score plot shows a clear differentiation among the molecules. In particular, sucrose and fructose appear to be well differentiated from the other three sugars along PC1 and PC2, respectively. By looking at the corresponding loadings, this can be interpreted by postulating that sucrose has a more pronounced minimum in the current trend at around 300 s if compared to the other molecules. On the other hand, the loadings on PC2 suggest that fructose has high contributions to a current trend and a low current drop.

**Figure 4:**
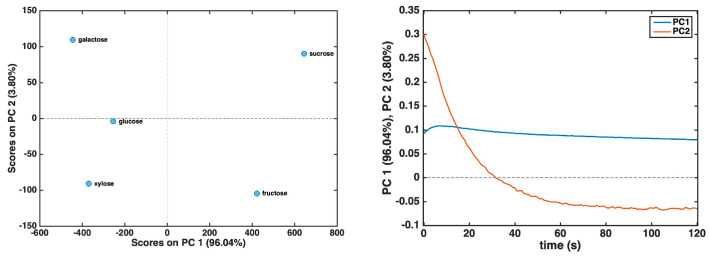
PCA results for the dataset comprising current trends up to 120 s: score plot (**left** panel); loading plot (**right** panel).

However, recording the complete response curves for each of the five carbohydrates up to 4000 s required a considerable amount of experimental time, which, in our view, made the analysis process excessively lengthy. Based on PCA evidence indicating that the most significant part of the current trends occurred at the beginning, we considered accelerating the analysis by focusing only on the first two minutes of the response curve (i.e., the initial points up to 120 s). Indeed, if successful, this approach would significantly reduce both analysis time and costs. Accordingly, a new PCA model was developed using the reduced dataset, which consisted of current trends up to 120 s after mean centering. The resulting score and loading plots, relative to the first two principal components—accounting for more than 99% of the original variance—are presented in Figure 4.

By examining the score plot in Figure 4, it becomes evident that the five sugars are more distinctly separated compared to the model constructed using the entire response curve. More specifically, PC1 differentiates fructose and sucrose from the other three carbohydrates. The inspection of the loading plot suggests that xylose, galactose, and glucose exhibit a trend where the current reaches a steady state more rapidly within the first 30 s of decay. In contrast, loadings on PC2 are associated with a slower current evolution, with a higher contribution observed for sucrose and galactose and a lower contribution for xylose and glucose.

Additionally, considering that the initial portion of the current trends follows a quasi-linear behavior, the corresponding current vs. time data points were fitted using a univariate regression approach. The results of this analysis are summarized in Table 1.

**Figure 5:**
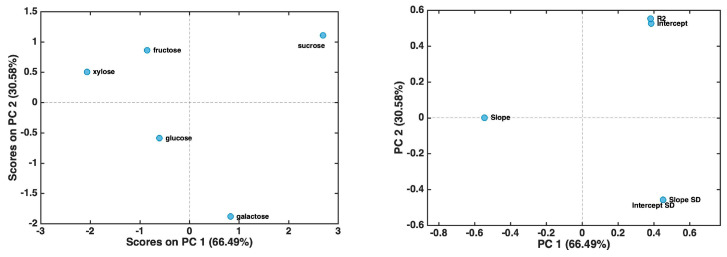
PCA results based on the straight-line fitting data summarized in Table 1: score plot (**left** panel); loading plot (**right** panel).

The data reported in Table 1 were used to calculate a further PCA model to differentiate the five investigated carbohydrates and the corresponding score and loading plots along the first two components, explaining more than 97% of the original variance; they are displayed in Figure 5.

As in the previous case, the five sugars are well separated from one another. Specifically, the loadings along the two principal components indicate that PC1 is primarily influenced by the slope of the regression lines, with xylose exhibiting the highest slope and galactose and sucrose the lowest. Among them, sucrose appears to have the best linear fit, whereas galactose shows higher uncertainty in estimating the two regression parameters.

**Figure 6:**
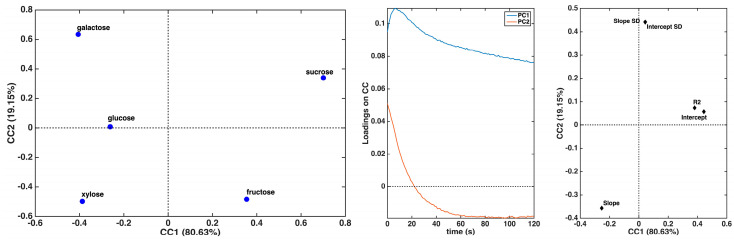
Results of data fusion by means of the ComDim algorithm: scores of the five analyzed carbohydrates relative to the first two common components (**left** panel); loadings of the current trends (**central** panel); loadings of the regression parameters (**right** panel).

Finally, to gain deeper insights and achieve a more comprehensive differentiation of the investigated carbohydrates, the current trends (up to 120 s) and the straight-line fitting parameters were jointly analyzed using a multi-block exploratory approach with the ComDim algorithm. The sample scores along with the first two common components, which account for more than 99% of the total variance, and the loadings for the two data blocks are reported in Figure 6.

The score plot shows that all five sugars can be differentiated from one another. More specifically, fructose and sucrose are separated from the other three along the first common component, while the second common component provides a further differentiation (between fructose and sucrose and among xylose, glucose, and galactose). The inspection of the block salience for the first two components evidences that the first component explains more than 90% of the variance of the current trend block and around 40% of the variance of the univariate regression parameter block. On the other hand, the second component accounts for about 5% of the variance in the current curve block and 53% of the variance in the regression parameter table block.

Overall, the inspection of the loadings suggests that, as far as the linear regression parameters are concerned, differentiation is governed by a contrast between the values of the slope and those of the intercept, the latter being also highly correlated with R2. At the same time, the uncertainty on the estimates of slope and intercept seems to have a high impact on the second component, therefore being the highest for galactose and lowest for fructose and xylose. When examining the loadings on the current curves, the observations align with those previously discussed when analyzing the current trends alone. The two components correspond to processes with different rate constants. Specifically, fructose and sucrose exhibit a more pronounced variation in current intensity during the early stages of the curve compared to the other three sugars. Conversely, the second component is associated with a distinct variation trend observed for galactose and sucrose.

## 4. Potential for Quantitative Analysis

As demonstrated in previous studies [11], it is also possible to perform quantitative analyses for individual carbohydrates by constructing and utilizing specific calibration curves based on increasing concentrations of each carbohydrate. Each calibration curve will have a distinct slope value depending on the carbohydrate being analyzed (see Table 2).

It is interesting to note that sucrose exhibits the highest slope value, as it also shows a higher current response at the same concentrations compared to the other carbohydrates. This can likely be attributed to the fact that sucrose is a disaccharide, unlike the other four monosaccharides. This observation suggests that the enzymatic system involved in yeast metabolism is capable of hydrolyzing sucrose into its respective monosaccharides.

## 5. Discussion

Instrumental chemical methods for the qualitative identification of organic substances are well established. Techniques such as chromatography, particularly when coupled with mass spectrometry (MS) [22,23], and nuclear magnetic resonance (NMR) spectroscopy [24,25] offer powerful analytical capabilities. However, chromatographic methods require significant time for application and optimization, while NMR- and MS-based approaches demand expensive equipment and expertise. In contrast, the method described in this study requires only low-cost equipment. Modern catalytic fuel cells are commercially available at minimal cost, and even their manual assembly by users does not require specialized skills. Furthermore, the simple instrumentation outlined in this work can be used not only for qualitative analyses but also for quantitative determinations. This is achieved by constructing conventional calibration curves that correlate the steady-state current values with increasing concentrations of the target substance.

Given its accessibility and ease of use, we believe that this method will appeal to a wide range of researchers and practitioners. As emphasized in the Introduction, our research group has recently focused on exploring the analytical potential of catalytic fuel cells, particularly for methanol and ethanol analyses. This includes both commercially available direct catalytic methanol or ethanol fuel cells (DC(M or E)FC) and their application in detecting other aliphatic alcohols in real matrices. Initially, our efforts were directed toward quantitative analytical applications [7,8,9]. However, more recently, we have explored the feasibility of qualitative analysis [10] using advanced chemometric methods to process the experimental data obtained from fuel cells. Typically, fuel cell response studies utilize the entire discharge curve generated upon introducing an alcoholic fuel—such as ethanol, methanol, or other aliphatic alcohols—into the system. However, our recent work [10] has highlighted the potential significance of charging curves in qualitative analysis. These curves may even contain more relevant information than the discharge curves in some cases. It is now evident that the qualitative information embedded in the charging curves inevitably influences the discharge curves as well. The key challenge lies in optimizing the use of charge and discharge curves for qualitative analyses. To achieve this, selecting an appropriate chemometric method for processing the data obtained from these curves is crucial.

## 6. Conclusions

In this study, we investigated two critical aspects related to our recent research in this field.

The first major objective was to optimize the amount of data required from fuel cell response curves and refine the chemometric data processing approaches. Our findings indicate that analysis times can be significantly reduced by utilizing only the initial segment of the response curves (up to 120 s). Additionally, a multi-block exploratory approach using the ComDim algorithm proved to be the most effective chemometric method for this purpose, delivering optimal results.

Another key aspect of this research was demonstrating that the proposed qualitative analysis method is highly effective not only for direct analysis of aliphatic alcohols [10] but also for cases where non-alcoholic fuels must first be transformed into alcohols. For example, carbohydrates can be converted into alcohols through yeast-based biological processes [11]. Our findings confirm that fuel cell discharge curves retain qualitative information about the original fuel, even if it is not an alcohol. This allows for the successful qualitative (and quantitative [11]) analysis of these organic compounds despite their necessary prior conversion into alcohols.

Finally, we aim to extend this fuel cell-based recognition method—both qualitative and quantitative—to other organic substances. This includes compounds that, while not classified as alcohols, contain at least one hydroxyl (-OH) group, with at least partially alcoholic properties, and natural polymeric substances that can be enzymatically or fermentatively converted into alcohols. Additionally, we will continue exploring alternative chemometric methods to enhance data processing, though our current approach has already demonstrated a high level of reliability and maturity.

## Figures and Tables

**Table 1:** Results of the linear fitting applied to data recorded from the five studied carbohydrate solutions, all at the same concentration (30 mmol L^−1^) and incubation time (12 h), using only the initial points of the curves (up to 120 s).

Carbohydrate	Slope	Slope SD	Intercept	Intercept SD	R^2^
**Sucrose**	−1.9970	0.1453	335.77	10.09	0.6134
**Fructose**	−1.4883	0.1254	286.67	8.71	0.5419
**Glucose**	−1.5602	0.1355	229.02	9.41	0.5269
**Galactose**	−1.7417	0.153	221.94	10.62	0.5213
**Xylose**	−1.4019	0.1187	209.56	8.24	0.5396

**Table 2:** Slope values (µA/mol L^−1^) of the calibration curves as a function of increasing concentrations of the examined carbohydrates (within the concentration range of 0.00 to 0.03 mol L^−1^).

Carbohydrates	Glucose	Xylose	Fructose	Galactose	Sucrose
**Slope values**	6865.3	6607.7	7915.3	7056.7	10,353

## Data Availability

All experimental data are available in the Supplementary Materials and in the text of this paper.

## Supplementary Materials

The following supporting information can be downloaded at https://www.mdpi.com/article/10.3390/bios15020096/s1. Table S1: Experimental data of the complete current response profile (up to 4000 s) recorded for the five analyzed carbohydrate samples.

## References

[1] Munteanu I.G., Apetrei C. (2022). A Review on Electrochemical Sensors and Biosensors Used in Assessing Antioxidant Activity. Antioxidants.

[2] Katey B., Voiculescu I., Penkova A.N., Untaroiu A. (2023). A Review of Biosensors and Their Applications. ASME Open J. Eng..

[3] Wu J., Liu H., Chen W., Ma B., Ju H. (2023). Device integration of electrochemical biosensors. Nat. Rev. Bioeng..

[4] Ali J., Najeeb J., Ali M.A., Aslam M.A., Raza A. (2017). Biosensors: Their Fundamentals, Designs, Types and Most Recent Impactful Applications: A Review. J. Biosens. Bioelectron..

[5] Elugoke S.E., Adekunle A.S., Fayemi O.E., Akpan E.D., Mamba B.B., Sherif E.S.M., Ebenso E.E. (2020). Molecularly imprinted polymers (MIPs) based electrochemical sensors for the determination of catecholamine neurotransmitters—Review. Electrochem. Sci. Adv..

[6] Campanella L., Sammartino M.P., Tomassetti M. (1992). New enzyme sensor for phenol determination in non-aqueous and aqueous medium. Sens. Actuators B.

[7] Tomassetti M., Angeloni R., Merola G., Castrucci M., Campanella L. (2016). Catalytic fuel cells used as an analytical tool for methanol and ethanol determination. Application to ethanol determination in alcoholic beverages. Electrochem. Acta.

[8] Tomassetti M., Angeloni R., Castrucci M., Martini E., Campanella L. (2018). Ethanol content determination in hard liquor drinks, beers, and wines, using a catalytic fuel cell. Comparison with other two conventional enzymatic biosensors: Correlation and statistical data. Acta Imeko.

[9] Tomassetti M., Merola G., Angeloni R., Marchiandi S., Campanella L. (2016). Further development on DMFC device used for analytical purpose: Real applications in the pharmaceutical field and possible in biological fluids. Anal. Bioanal. Chem..

[10] Tomassetti M., Marini F., Pezzilli R., Castrucci M., Di Natale C., Campanella L. (2024). Improvement of Qualitative Analyses of Aliphatic Alcohols Using Direct Catalytic Fuel Cell and Chemometric Analysis Format. Sensors.

[11] Tomassetti M., Dell’Aglio E., Castrucci M., Sammartino M.P., Campanella L., Di Natale C. (2021). SimpleYeast-Direct Catalytic Fuel Cell Bio-Device: Analytical Results and Energetic Properties. Biosensors.

[12] Tomassetti M., Marini F., Angeloni R., Castrucci M., Campanella L., Di Natale C. (2020). Direct Catalytic Fuel Cell Device Coupled to Chemometric Methods to Detect Organic Compounds of Pharmaceutical and Biomedical Interest. Sensors.

[13] Zhang J., Zhang H., Wu J., Zhang J. (2013). Chapter 7—Fuel Cell Open Circuit Voltage. Pem Fuel Cell Testing and Diagnosis.

[14] Sparks D., Laroche C., Tran N., Goetzinger D., Najafi N., Kawaguchi K. A new methanol concentration microsensor for improved DMFC performance. Proceedings of the 2005 Fuel Cell Summit.

[15] Pearson K. (1901). On lines and planes of closest fit to systems of points in space. Philos. Mag..

[16] Hotelling H. (1933). Analysis of a Complex of Statistical Variables into Principal Components. J. Educ. Psychol..

[17] Wold S., Esbensen K., Geladi P. (1987). Principal component analysis. Chemom. Intell. Lab. Syst..

[18] Jolliffe I.T. (2002). Principal Component Analysis.

[19] Qannari E.M., Wakeling I., Courcoux P., MacFie H.J.H. (2000). Defining the underlying sensory dimensions. Food Qual. Prefer..

[20] Cariou V., Qannari E.M., Rutledge D.N., Vigneau E. (2018). ComDim: From multiblock data analysis to path modeling. Food Qual. Prefer..

[21] Jouan-Rimbaud Bouveresse D., Rutledge D.N. (2024). A synthetic review of some recent extensions of ComDim. J. Chemom..

[22] Badawy M.E.I., El-Nouby M.A.M., Kimani P.K., Lim L.W., Rabea E.I. (2022). A review of the modern principles and applications of solid-phase extraction techniques in chromatographic analysis. Anal. Sci..

[23] Ranjan Maji S., Roy C., Kumar Sinha S. (2023). Gas chromatography–mass spectrometry (GC-MS): A comprehensive review of synergistic combinations and their applications in the past two decades. J. Anal. Sci. Appl. Biotechnol..

[24] Pismennõi D., Kiritsenko V., Marhivka J., Kütt M.-L., Vilu R. (2021). Development and Optimisation of HILIC-LC-MS Method for Determination of Carbohydrates in Fermentation Samples. Molecules.

[25] Mitschke N., Vemulapalli S.P.B., Dittmar T. (2023). NMR spectroscopy of dissolved organic matter: A review. Environ. Chem. Lett..

